# Cochlear Implant Results in Older Adults with Post-Lingual Deafness: The Role of “Top-Down” Neurocognitive Mechanisms

**DOI:** 10.3390/ijerph19031343

**Published:** 2022-01-25

**Authors:** Milena Zucca, Andrea Albera, Roberto Albera, Carla Montuschi, Beatrice Della Gatta, Andrea Canale, Innocenzo Rainero

**Affiliations:** 1 Department of Neuroscience, Aging Brain and Memory Clinic, University of Torino, 10126 Turin, Italy; innocenzo.rainero@unito.it; 2 Department of Surgical Sciences, ENT Division, University of Torino, 10126 Turin, Italy; aalbera@hotmail.com (A.A.); roberto.albera@unito.it (R.A.); beatrice.dellagatta@unito.it (B.D.G.); andrea.canale@unito.it (A.C.); 3 Section of Otorhinolaryngology, Department of Surgery, Ospedale degli Infermi, 13875 Biella, Italy; carla.montuschi@aslbi.piemonte.it

**Keywords:** cochlear implant, hearing impairment, older adults, neurocognition, processing speed, speech recognition

## Abstract

To date, no clear specific cognitive predictors of speech perception outcome in older adult cochlear implant (CI) users have yet emerged. The aim of this prospective study was to increase knowledge on cognitive and clinical predictors of the audiological outcome in adult cochlear implant users. A total of 21 patients with post-lingual deafness, who were candidates for cochlear implantation, were recruited at the Department of Ear, Nose and Throat, University of Torino (Italy) and subjected to a pre-operatory neuropsychological assessment (T0) and an audiological examination after 12 months of implantation (T12). Patients who, at T12, had a 60 dB verbal recognition above 80%, were younger (z = −2.131, *p* = 0.033) and performed better in the Verbal Semantic Fluency Test at T0 (z = −1.941, *p* = 0.052) than subjects who had a 60 dB verbal recognition at T12 below 80%. The most significant predictors of the CI audiological outcome at T12 were age (β = −0.492, *p* = 0.024) and patients’ TMT-A performance at baseline (β = −0.486, *p* = 0.035). We conclude that cognitive processing speed might be a good predictor of the level of speech understanding in older adult patients with CI after one year of implantation.

## 1. Introduction

Hearing impairment is the third most prevalent chronic medical condition among older adults, affecting one in five people over fifty years of age [1]. Several studies suggest that, in the elderly population, hearing deficit and cognitive impairment are strongly and independently associated [2], creating a two-way deleterious loop. Hearing impairment in older adults could be related with a 30–40% rate of increased cognitive decline [3,4] and higher risk of dementia [4,5,6,7]. Some authors have speculated that the relationship between hearing impairments and cognitive deficits can be explained by the “effortfulness hypothesis” [1,8]. According to this hypothesis, in order to better understand speech, patients with deafness tend to put more pressure on their limited higher-order neurocognitive resources and “top-down” mechanisms, with a substantial deterioration of their cognitive performance [9]. On the other hand, an age-related decline in cognitive abilities (i.e., reduction in attentional resources, working memory capacity, processing speed, etc.) can affect the ability of older adults to comprehend and remember fast-paced speech in daily life [1].

Cochlear implant (CI) is the treatment of choice for profound deafness hearing rehabilitation [10]. In the past few years, significant improvements have been obtained in audiological outcomes due to technological advancements.

However, despite these very good results with regards to its application, a wide intersubjective variability in the perception of language in CI older adult users [11] has been observed. The cochlear implantation outcome can be affected by multiple factors, such as the physical characteristics of the device (number of electrodes, frequency range, compression of the loudness dynamics, interaction between electrodes), the positioning of the device (depth of insertion, respect of tonotopy) and the clinical features of the implanted subject (age, date of onset and duration of deafness, cause of deafness and number of residual fibers of the auditory nerve) [12,13,14,15].

It is also well-known that speech intelligibility depends on two factors: an extrinsic one, linked to the redundancy of the verbal message, and an intrinsic one, which requires a constant involvement of “top-down” neurocognitive mechanisms and executive functions [16], and is linked to the ability of the central auditory processing system to decipher the spectral, spatial and temporal properties of sound [17].

To date, no clear specific cognitive predictors of speech perception outcome in CI users have emerged, and available data, provided only by a few studies [18,19,20,21,22,23,24,25], are still scarce and generally inconsistent.

Although most of these studies speculate that cognitive abilities can play a role in the speech recognition of CI users [18,19,21,22,23,24,25], not all results are in line with this assumption [20].

Furthermore, there is still disagreement on which higher cognitive abilities, among those evaluated, are involved in the audiometric outcomes of CI. For some authors, global working memory capacities [19], cognitive processing speed [18,19] and inhibition skills [21,24] might be cognitive abilities that are critically involved in audiological outcomes after implantation. Other studies, instead, report that clinical speech recognition outcomes for adult implant users mostly relate with phonological capacities [23] or specific components of the working memory [24,25].

Finally, most studies use different auditory tasks to assess the same neurocognitive functions and only a few of these are prospective (i.e., have correlated preoperative cognitive measures with postoperative listening performance) [18,19,20,22,25], while others are case-controls research [21,23,24]. This variability in methodology may have increased the risk of bias due to the impaired audibility of patients, and can partially explain the heterogeneous and poorly consistent findings of these research studies.

The purpose of this prospective study was, therefore, to understand the possible predictive role of cognitive abilities on the quality of audiological outcomes after one year of implantation in a sample of post-lingual adult subjects who were candidates for CI.

This hypothesis was evaluated using a comprehensive pre-operatory and non-auditory neurocognitive protocol that included different tests for the assessment of both the general cognitive functioning and specific cognitive domains, that could be related with language understanding, such as verbal learning, verbal short term and working memory, visuospatial short term and working memory, language, executive functioning abilities, divided attention, cognitive processing speed and cognitive shifting.

In addition, we aimed at expanding knowledge on the predictive impact of clinical characteristics (age, aetiology, deafness duration, implant side of auditory deprivation, binaural hearing rehabilitation) on the audiological results in this population.

## 2. Materials and Methods

### 2.1. Sample and Setting

This is a prospective study including 21 patients (M/F = 10/11; mean age ± SD = 65 ± 8 years) with post-lingual deafness who were recruited at the Department of Ear, Nose and Throat, University of Torino (Italy) and were implanted with a CI between July 2016 and November 2018. Participants were included in the study if they had post-lingual deafness, were aged 50 or above at the onset of deafness, and if they were eligible for cochlear implant according to the existing Italian guidelines [26,27]. Preoperative CT and MR show a patent cochlea in all patients. Written informed consent was obtained from all of the participants, and the study was approved by the Hospital Ethics Committee.

The demographic and clinical characteristics of the patients involved in the study are shown in Table 1.

In case of asymmetric hearing loss, the CI was positioned in the worse ear, according to the literature about acquired hearing loss [28]. In 15 patients (71%), hearing rehabilitation consisted of bimodal stimulation with the CI in the worse ear, and a hearing aid (HA) in the contralateral ear. In the better ear, pure tone audiometry at each frequency (500–1000–2000–4000 Hz) was worse than 65 dB. The speech recognition score (SRS) at 60 dB (see audiological examination section for details) with only the HA fitted was worse than 50%. Table 2 shows the audiometric data of the aided ears.

Cochlear implants were positioned on the right side in 61.9% of patients and on the left side in 38.1%. The average deafness duration was 23.8 years (SD = ±13.7 years). Moreover, 46% of the study population had been suffering from hearing loss for over 20 years, 57.2% for 20 years or less. The average auditory deprivation in the implanted side was 8.6 years. 33% of the study population had been suffering from auditory deprivation in the implanted side for more than 15 years, 66.7% for less than 15 years. Hearing loss was caused by sudden hearing loss (19%), idiopathic factors (33%), iatrogenic factors (5%), meningitis (5%), Menière Disease (10%), otosclerosis (19%), autoimmune conditions (9%). Prior to implantation, patients underwent an extensive neuropsychological preoperative assessment (T0). The neuropsychological session took approximately 60 min. Audiological examination, measuring the patient’s verbal recognition at 60 dB, was performed 12 months after cochlear implant activation (T12).

### 2.2. Measurements and Instruments

#### 2.2.1. Neuropsychological Assessment

The neuropsychological evaluation at T0 was carried out by assessing the global cognitive functioning using the Mini-Mental State Examination (MMSE) [29] and the Clock Drawing Tests (CDT) [30,31]. Specific cognitive domains were assessed with the use of different scales: verbal learning and memory with the Rey 15 Words Auditory Learning Test (RAVLT) [32]; verbal short term and working memory with the Digit-Span Test [33,34,35]; visuospatial short term and working memory with the Corsi Block-tapping Test [36,37]; language and executive functioning abilities with the Verbal Fluency Test [37,38]; divided attention, cognitive processing speed and cognitive shifting with the Trial Making Test (TMT) [37,39]. In the TMT test, higher scores correspond to a worse performance. The Verbal Fluency Test version used for this study included a phonemic fluency task (using the letters FAS) and a semantic fluency task that requires the naming of colours, animals, fruits, and cities.

#### 2.2.2. Stimuli-Specific Procedures

Neuropsychological assessment was performed using a PowerPoint presentation shown on a laptop. Patients were requested to read the instructions aloud from a computer screen, so that the examiner could ascertain if the subject had properly understood the instructions of the test. In this way, patients were all tested in the same reliable way, thus reducing the auditory bias of the evaluation and variability between subjects. In addition, the stimuli in the MMSE, Digit Span, and RAVLT tests were also visually presented. In the RAVLT test, words were presented in uppercase letters. Each item was visible to the subject for 1.25 s, followed by a 0.75 s interval between the items at a 2 s rate for each word. Finally, in the Digit Span test numbers were presented at a 1.50 s rate. Within the 1.50 s period, the number was visible to the subject for 1.00 s, followed by a 0.50 s interval.

#### 2.2.3. Audiological Examination

All of the patients were evaluated with free field pure tone audiometry with the CI at frequencies between 125 Hz and 8000 Hz to verify that the audiometric threshold was balanced in terms of loudness between electrodes. The PTA of all frequencies was ≤25 dB.

Subsequently, all of the patients underwent free field speech audiometry. Speech recognition scores (SRS) were obtained performing the speech test suggested by the Italian Guidelines for cochlear implant indications at 60 dB [10]. A total of 20 meaningful disyllabic words in an open set, phonetically balanced, pronounced by a recorded female voice cadenced every 3.5 s. were delivered through a loudspeaker at 0 degrees Azimuth in the sound field.

The lists were extracted from one of the tests that, at the time of the evaluation, were most commonly used to assess speech intelligibility in the Italian language [40].

Distinct from the speech test suggested by the Italian CI Guidelines, 2 lists (10 words each) were submitted in a quiet environment, without a signal to noise ratio (SNR) +10. The aim of using two lists was to increase the accuracy of the results. Sentences or words in noise were not used to minimize variation of the outcome due to extra auditory ability.

The study group was divided between patients with favourable prognosis and patients with unfavourable prognosis for the outcome, on the basis of the existing literature [11,41]. Patients 4, 7, 8, 11, 12 and 13 were considered patients with unfavourable prognosis because their aetiology was, respectively, chronic otitis media, otosclerosis, infantile meningitis and otosclerosis for the last three patients.

#### 2.2.4. Statistical Analysis

Statistical analysis was performed using SPSS-version 21.0 for Windows (IBM SPSS Statistics, Inc., Chicago, IL, USA). The 60 dB verbal recognition outcome at T12 was compared between group pairs using the Mann-Whitney U test, after stratifying the sample according to (1) type of prognosis (favourable or unfavourable) (2) gender (3) duration of deafness (≤20 or >20 years) at T0 and (4) presence or absence of a Binaural Hearing Rehabilitation.

In addition, to perform the statistical analysis a cut-off value delimiting good audiological performance was chosen. Based on the mean CI results found in the literature, the cut-off value was identified at SRS > 80% [11]. Using the Mann-Whitney U test, cognitive abilities, age and educational level at T0 were compared between patients with a percentage of 60 dB verbal recognition at T12 > 80% and subjects with a percentage of 60 dB verbal recognition at T12 ≤ 80%.

To evaluate the correlations between cognitive abilities at T0, the clinical characteristics of patients, and verbal recognition at 60 dB at T12 in the general population, a series of linear regression models were performed for each independent variable. The 60 dB verbal recognition at T12 was considered as a dependent variable. As independent variables, we took in consideration the different clinical features and the results of the tests used to assess the patients’ preoperative cognitive performance. The results were presented as Odds Ratio (OR) with a 95% confidence interval. All of the tests were performed as two-sided, *p* < 0.05 stands for statistically significant value.

## 3. Results

### 3.1. Relation between CI Audiological Outcome at T12 and Clinical Features of Patients after Stratification of Sample by Various Characteristics of Interest

In the general population, the mean of speech recognition score at T12 was 81.6% (SD = ±21.5). The number of subjects with a score >80% was 14 (66.6%).

After a stratification of the patients’ cohort according to specific clinical characteristics of interest, no differences were found in respect to the percentage of 60 dB verbal recognition at T12 between: (1) patients with a favourable prognosis and patients with an unfavourable prognosis, with reference to their aetiology; (2) male or female gender; (3) subjects with a duration of deafness ≤20 or >20 years at T0; (4) patients with or without a Binaural Hearing Rehabilitation. Patients that had a 60 dB verbal recognition at T12 above 80% were younger (z= −2.131, *p* = 0.033) than subjects that had a 60 dB verbal recognition at T12 below 80%

### 3.2. Identification of Clinical Predictors of CI Audiological Outcome at T12 through Linear Regression Analyses

**Figure 1:**
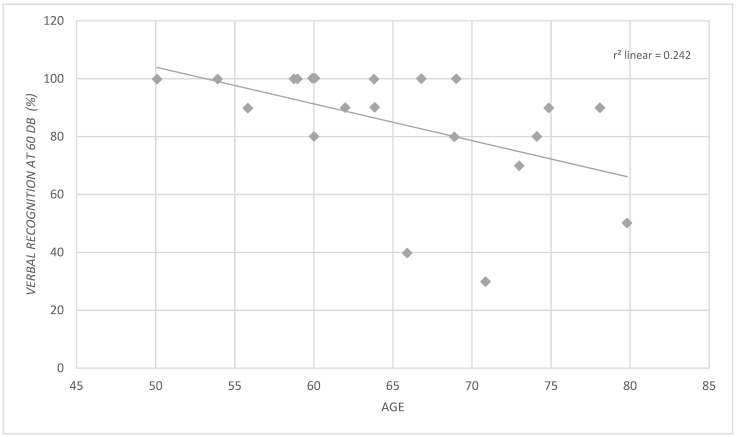
Scatter plot showing the significant correlation between age and verbal recognition at 60 dB after one year from the cochlear implantation.

In the general population, linear regression analyses showed that age (β = −0.492, *p* = 0.024) was the most significant clinical predictor of the CI audiological outcome at T12 (Figure 1).

### 3.3. Relation between CI Audiological Outcome at T12 and Cognitive Abilities at T0 after Stratification of the Patients’ Cohort Based on Percentage of CI Verbal Recognition at One Year of Implantation

Table 3 shows the difference in cognitive results at T0 between patients with a percentage of 60 dB verbal recognition >80% and subjects with a percentage of 60 dB verbal recognition ≤80% after one year of implantation. No significant differences in cognitive performance at baseline were found between the two groups. Only a trend in the Verbal Semantic Fluency Test outcome at T0 (z= −1.941, *p* = 0.052) has emerged: subjects that had a 60 dB verbal recognition at T12 above 80% performed better in the Verbal Semantic Fluency Test at T0 than subjects that had a 60 dB verbal recognition at T12 below 80%.

### 3.4. Identification of Cognitive Predictors of CI Audiological Outcome at T12 through Linear Regression Analyses

In the general population, linear regression analyses showed that the TMT- A performance of patients at T0 (β = −0.486, *p* = 0.035) was the most significant cognitive predictor of the CI audiological outcome at T12.

Correlations emerged from linear regression analyses between cognitive performances at T0 and audiological outcome after one year of cochlear implantation are shown in Table 4.

## 4. Discussion

Our study showed that the audiological outcome, measured as 60 dB verbal recognition 12 months after cochlear implant activation, is related both to different clinical features and to pre-operative cognitive assessment results. An analysis of clinical and cognitive features across subgroups of participants, stratified by various characteristics of interest, and regression analyses performed in our study, confirms the relationship between hearing loss, quality of audiological outcomes and cognitive abilities, outlining some interesting associations between these aspects.

### 4.1. Clinical Characteristics and Predictors

Aetiology of hearing impairment, duration of deafness, gender and presence or absence of a Binaural Hearing Rehabilitation did not seem to affect audiological outcomes, even in case of unfavourable aetiology, such as meningitis and otosclerosis. It is reported that aetiology could affect audiological outcomes [11,14,41,42,43,44]. Meningitis, otosclerosis and chronic otitis media present a risk of partial cochlear ossification that does not contraindicate the insertion of electrodes but could reduce the hearing outcome. According to the recent literature, duration of deafness does not seem to significantly affect the hearing outcome in case of acquired hearing loss [28,45].

In our sample, we have not found a correlation between auditory results and bimodal stimulation. Our results are apparently in contrast with the literature [46]. However, it is important to note that our aim was not to emphasize the properties of binaural hearing and we have not performed speech tests in noise with settings aimed at investigating the squelch effect or head shadow effect. Moreover, the acoustically stimulated ears in our study obtained low speech recognition scores, with a mean value of 25%. For this reason, it is possible that the contribution of the HA for speech results in this audiometric setting, characterized by quiet conditions, is negligible. The considerable number of patients with maximum SRS (9/21) is therefore due to the speech test used. As previously mentioned, a test without noise was performed to reduce extra auditory confounding factors.

Analysing the clinical characteristics of our sample, we found that patients that had a 60 dB verbal recognition at T12 above 80% were younger than subjects with a 60 dB verbal recognition at T12 below 80%. Age is also the most significant clinical predictor of the CI audiological outcome at T12 that emerged from regression analyses in the general population.

Different examples of how elderly age can negatively affect audiological outcomes are reported in the literature. While some authors only describe audiological performances worsening in noisy conditions [44], others describe a general worsening of vocal recognition as age increases [47]. Nevertheless, cochlear implantation has no age limitations, due to the significant improvement in life quality reported by implanted elderly patients [48,49,50].

### 4.2. Cognitive Abilities and Predictors

In our sample, the TMT-A performances of patients at T0 represent the most predictive cognitive variable among the neurocognitive abilities evaluated at baseline for the audiological outcome at T12. It is widely known that performance in the TMT-A test provides an index of processing speed [51], which progressively declines with age [52]. This is also demonstrated by the close relation found between performance exhibited in the TMT tasks and age-related cognitive deficits [53]. Previous studies reported that language comprehension and production are affected by the slowdown of information processing, which is also related to impairments in working memory and attention [54]. In addition, the quality of TMT performance appears to predict the speech perception in listeners with and without hearing loss [55,56,57]. Intriguingly, other prospective studies [18,19] also speculated that speed of response and cognitive processing speed might be critically important predictors of speech recognition quality in CI users after 6, 8 and 18 months of implantation.

Our data, however, are in contrast with another study [22], which does not find any association between the performance in the TMT-A test exhibited by seven elderly female CI users at pre-operatory assessment and quality of word recognition one or more years after implantation. However, it is important to note that, in their research, Cosetti et al. [22] only used cognitive tests that significantly improve after implantation to predict the post-CI speech perception outcome. Heydebrand et al. [20] have found that verbal learning and lipreading skills, but not processing speed, were significantly correlated with audiological outcome at 6 months in adult patients with CI. It is important to note that these authors, in order to assess processing speed, used a methodology that included tests that required to discriminate between letters and words, with a greater involvement of language skills. This may have influenced their results and can partially explain the discrepancy found with our data.

### 4.3. Processing Speed and Quality of Audiological Outcome in Older Adult CI Users: A New Hypothesis Based on a Multi-Modal Model for Language Understanding

A possible explanation of our results can be provided by one of the most influential and comprehensive models of language comprehension proposed by Rönnberg et al. [16]: the Ease of Language Understanding (ELU) model. This model postulates that a slower top-down processing is involved when the perceived signal-due to a degradation of language input or a reduction in processing speed-fails to match with a corresponding syllabic phonological representation stored in the semantic long-term memory. These top-down mechanisms require the use of specific executive cognitive resources, such as the working memory (WM), i.e., a storage system with a limited capacity where we hold and manipulate information over short periods of time [58]. The ability of the WM to maintain information is partly determined by the speed of cleaning the memory storage between tasks: the higher the refresh rate of this memory warehouse, the greater the capacity of the WM. However, as reported by McCabe et al. [59], although processing speed appears to be robustly correlated with WM capacity, it is nonetheless distinct from it.

In line with these suggestions, and in accordance with what has already been hypothesized by other researchers [60,61,62], our assumption is that, in adult and elderly CI patients, a reduction in cognitive processing speed in the top-down processes involved in case of mismatch between the perceived language signal and its representation in the semantic LTM can affect the ability of the WM, which is limited by a slower refresh rate of the memory trace. Therefore, a reduction in WM capacity can unfavourably influence the controlled retrieval of long-term semantic knowledge and, consequently, the audiological outcomes of cochlear implant users.

Our hypothesis is also supported by the fact that, in our sample, we have found that subjects with a 60 dB verbal recognition at T12 above 80% performed better in the Verbal Semantic Fluency Test at T0 than patients with a 60 dB verbal recognition at T12 below 80%. Interestingly, another study [23] previously found that audiological outcomes in CI users are strongly related to their capacity to process phonological information. In addition, data obtained from neuroimaging examinations [63] confirm that the main brain areas involved in the quality of auditory recovery, including in CI users, are located outside the auditory cortex. They predominantly involve prefrontal regions, such as the left inferior prefrontal cortex, which is involved in the control of semantic information recovery and in the evaluation of meaning [64]. In the ELU model, smooth access to semantic representations in the long-term memory is very important for good language comprehension, especially in case of mismatch [16]. It was also reported that a general reduction in cognitive speed affects verbal fluency much more than a circumscribed deficit in executive functioning [65].

In accordance with previous studies [18], we hypothesize that processing speed can play a significant role at the bottom-up level as a risk factor, in combination with other parameters, during the mismatch phase. Subsequently, it can influence different cognitive and executive skills (in addition to WM) in the top-down processes occurring after the mismatch. These findings could probably be explained by the fact that, in contrast to other studies [19], in our research we have not identified the WM as a specific predictive variable. It is indeed possible that, in an older CI population, a general deterioration in processing speed plays a more significant role in the quality of audiological outcomes in respect to individual executive abilities, such as the working memory.

Our study has some limitations that should be acknowledged. Firstly, we have only used the raw scores obtained by patients in the cognitive tests at baseline. However, there has not been any validation of the neuropsychological tests (including TMT) of the ARHI population yet. The use of correct, standardized scores in this specific research settings can therefore be misleading. The choice of a better protocol to assess the cognitive profile of patients with hearing loss, hearing aids or cochlear implants is certainly an aspect that should be seriously taken into consideration in both clinical and research settings to ensure a greater accuracy and reliability of results. Our study highlights this limitation and emphasizes the need to validate a standardized and specific neuropsychological protocol for these patients to increase scientific knowledge and reduce the bias of their cognitive clinical evaluation.

Another limitation of the present study is the fact that we have assessed audiometric outcomes only at one year after implantation, and not at two years or later, similarly to how it was conducted in another study [22]. This choice was made because, in our experience, hearing results greatly improve in the first six months of speech therapy rehabilitation, peaking after one year [28,43].

Thirdly, our patients were subjected to cognitive evaluation only prior to cochlear implantation. Although a follow-up evaluation could have consolidated our data, it is important to note that our research was specifically focused on understanding which cognitive abilities at baseline, without any kind of rehabilitation or stimulation, can predict the quality of audiological outcomes after one year of implantation. In addition, this methodology has already been used by other authors [18,20].

Finally, the study population is relatively small. The small size of our sample can probably explain why, after further dividing the patients according to the percentage of verbal recognition at one year of implantation, we found no significant differences, but only a trend in baseline cognitive performances between the two groups. It is, in fact, possible that this further subdivision of the sample has further reduced the statistical power of the analysis.

However, the sample size is in line with previous prospective research [18,19,20,21,22,23,24,25]. Furthermore, the patients recruited for the study were extensively investigated and also underwent a wide in-depth neuropsychological evaluation in the pre-operative stage.

## 5. Conclusions

Despite the limitations discussed above, we think that this prospective study offers an interesting perspective of approach to hearing problems, both from clinical and research point of view. First, our results partially confirm and bring new insights on the predictive impact of cognitive abilities on the audiological outcome in adult cochlear implant users with hearing impairments. Second, our data further support the presence of a two-way vicious circle of hearing impairment and cognitive decline. Finally, our data reinforce the need to include, especially with older subjects, a pre-operative standardized neurocognitive assessment, in order to give CI candidates realistic auditory expectations after implantation.

## Figures and Tables

**Table 1:** Clinical and demographic characteristics of patients.

Patient	Age (Years)	Education (Years)	Aetiology	IS	HL Duration (Years)	AD in IS (Years)	Controlateral HA
1	54	11	Sudden Hearing Loss	Right	10	3	Yes
2	69	18	Idiopatic	Right	8	0	No
3	73	5	Autoimmune	Right	2	0	Yes
4	56	9	Chronic Otitis Media	Right	30	30	Yes
5	71	12	Sudden Hearing Loss	Right	15	1	Yes
6	64	5	Idiopatic	Left	20	15	Yes
7	74	5	Otosclerosis	Left	40	4	Yes
8	50	18	Infantile Meningitis	Right	45	20	No
9	64	12	Idiopatic	Left	55	35	No
10	69	18	Idiopatic	Right	30	15	No
11	60	8	Otosclerosis	Left	25	4	Yes
12	66	8	Otosclerosis	Left	35	22	No
13	59	8	Otosclerosis	Right	40	15	No
14	78	18	Sudden Hearing Loss	Right	15	3	Yes
15	62	16	Idiopatic	Right	20	5	Yes
16	67	13	Idiopatic	Left	25	0	Yes
17	59	8	Autoimmune	Right	4	0	Yes
18	80	3	Menière Disease	Right	20	0	Yes
19	60	13	Sudden Hearing Loss	Left	15	1	Yes
20	75	8	Idiopatic	Right	20	3	Yes
21	60	13	Menière Disease	Left	25	4	Yes

Note. IS: implanted side; HL = hearing loss; AD = auditory deprivation; HA = hearing aid.

**Table 2:** Audiometric data of non-implanted ears in bimodal patients.

n	PTA Aided Side (dB)	SRS with HA (%)
1	101.25	10
3	93.75	20
4	92.5	20
5	100	10
6	91.25	10
7	70	50
11	78.75	30
14	101.25	10
15	73.75	40
16	90	10
17	71.25	50
18	101.25	10
19	71.25	50
20	106.25	10
21	76.25	50
Mean	87.92	25.33
SD	13.05	17.67

Note: PTA: pure tone average at 500–1000–2000–4000 Hz; SRS: speech recognition score; HA: hearing aid.

**Table 3:** Difference of cognitive results at T0 between patients with a percentage of 60 dB verbal recognition at T12 > 80% and subjects with a percentage of 60 dB verbal recognition at T12 ≤ 80% ^a^.

Cognitive Tests (T0)	Verbal Recognition at 60 dB > 80% (T12)	Verbal Recognition at 60 dB ≤ 80% (T12)	*p*-Value
MMSE	27.4 ± 2.3	25.6 ± 4.4	0.545
CDT	13.2 ± 1.7	10.6 ± 4.4	0.117
RAVLT **Immediate**	35.0 ± 8.0	30.5 ± 11.3	0.343
RAVLT **Differite**	6.7 ± 3.0	5.8 ± 4.3	0.455
Digit-Span Test **Forward**	5.2 ± 1.1	4.4 ± 1.3	0.199
Digit-Span Test **Backward**	4.1 ± 1.4	3.4 ± 1.1	0.382
Corsi block-tapping test **Forward**	5.1 ± 1.0	4.4 ± 0.8	0.220
Corsi block-tapping test **Backward**	4.3 ± 1.5	3.8 ± 1.3	0.588
Verbal phonemic Fluency Test	35.5 ± 12.0	30.1 ± 11.0	0.218
Verbal semantic Fluency Test	24.3 ± 4.0	19.1 ± 5.5	0.052
TMT-A	37.2 ± 18.4	51.8 ± 15.6	0.115
TMT-B	114.3 ± 71.9	241.8 ± 171.1	0.087
TMT B-A	77.3 ± 60.0	190.0 ± 157.6	0.138

Note. MMSE = Mini-Mental State Examination; CDT = Clock Drawing Test; RAVLT = Rey 15 Words Auditory Learning Test; TMT = Trial Making Test. In the TMT test, higher scores correspond to worse performance. ^a^ All data are presented as mean + SD.

**Table 4:** Correlations between cognitive results at T0 and verbal recognition at 60 dB after one year from the cochlear implantation.

Cognitve Tests (T0)	r^2^	β	*p*-Value
MMSE	0.061	0.247	0.280
CDT	0.177	0.421	0.058
RAVLT **Immediate**	0.049	0.222	0.346
RAVLT **Differite**	0.110	0.331	0.154
Digit-Span Test **Forward**	0.003	0.051	0.826
Digit-Span Test **Backward**	0.036	0.190	0.410
Corsi block-tapping test **Forward**	0.081	0.284	0.212
Corsi block-tapping test **Backward**	0.103	0.321	0.156
Verbal phonemic Fluency Test	0.002	0.049	0.834
Verbal semantic Fluency Test	0.165	0.407	0.067
TMT-A	0.236	−0.486	0.035
TMT-B	0.086	−0.370	0.119
TMT B-A	0.108	−0.328	0.170

Note. MMSE = Mini-Mental State Examination; CDT = Clock Drawing Test; RAVLT = Rey 15 Words Auditory Learning Test; TMT = Trial Making Test. In TMT test, higher scores correspond to worse performance.

## Data Availability

Data available on request due to restrictions eg privacy or ethical.
